# Irregular Menstruation in Young Women Due to Anemic Conditions

**Published:** 1897-12

**Authors:** H. Edwin Lewis

**Affiliations:** Burlington Vt.; Resident Physician Fanny Allen Hospital


### IRREGULAR MENSTRUATION IN YOUNG WOMEN DUE TO ANEMIC CONDITIONS.

By H. EDWIN LEWIS, M. D., Burlington Vt.

Resident Physician Fanny Allen Hospital.

The young physician just starting into practice can not help but be impressed with the frequent occurrence of menstrual disorders in young girls during the period just succeeding the age of puberty. The metamorphosis of a girl into a woman, consisting as it does of structural and functional changes throughout her body, in many instances leaves behind pronounced alterations in the quality or even quantity of the blood current. How common it is to have a mother bring her daughter to the physician and say, "Doctor, I would like to have you do something for my daughter. For nearly a year she has been losing interest in everything and seems to be completely worn out. She has no appetite and absolutely no ambition for work, study or play. She does not lose flesh or grow thin at all, but her color is so poor and she seems so weak that I fear she is going into consumption."

Inquiry on the part of the doctor elicits the further information that the young lady in question is sixteen years old or thereabouts, and that she is a schoolgirl. A year or two ago she first menstruated and since that time has been unwell only twice, or at irregular intervals varying anywhere from three to nine months. Her bowels are either constipated, or the reverse, and she may complain of headaches, vertigo, palpitation of the heart, insomnia, indigestion, etc., etc. The pale face with its sallow greenish tinge, the bleached tongue, the colorless conjunctivæ and finger nails, tell well the tale of impoverished blood. Combine the history with the objective symptoms and the diagnosis is clear of chlorosis or green-sickness. The absence of cough or pulmonary symptoms excludes the dreaded "consumption," but we have instead a condition of the blood in which the essential constituents are diminished and the whole quality of the life-giving current so depreciated that the various organs of the body are unable to perform their normal functions. The uterus is small and illy developed and the supply of rich blood it so urgently requires in its developmental state is not to be had. Is it any wonder, then, that the chlorotic girl does not menstruate regularly? It is a great wonder that she ever menstruates at all. Correct the anemic or impoverished condition of her blood and the physiological function of her uterus will be resumed as naturally as that of any other organ.

How this chlorotic condition can best be corrected is the next question and one which, because of its frequency, concerns every practicing physician. Countless remedies have been presented to the profession, but far and foremost above them all is iron, notwithstanding certain high authority to the contrary. Arsenic is certainly valuable but it ranks far below iron or even manganese in the therapeutics of anemia. In order to be most efficacious, however, the iron should be in its most readily assimilable form, and until recently the carbonate and albuminate have been supposed to present this requisite in the highest degree. But since manganese has grown in favor as an adjuvant to iron, a new preparation has been submitted to the medical profession *and in every way it has proven itself an ideal one.* I refer to Dr. Gude's preparation of the peptonate of iron and manganese, known as Pepto-Mangan. This admirable combination of iron and manganese is readily taken into the human economy and appropriated to its needs, without deranging the weakest alimentary tract, or hindering in any way the normal processes of digestion, assimilation and excretion. It should be given in water or milk in teaspoonful doses after meals, and its administration is invariably followed by the results desired.

But in order that the medical treatment of chlorosis may be most valuable and efficient, it should be augmented by auxiliary treatment consisting of careful attention to diet and exercise. It goes without saying that the food of an anemic girl should be most nutritious and particularly abundant in albumen, while the exercise should aim to provide greater quantities of oxygen in the form of pure air, without lowering the vitality. Walking, skating, tennis or bicycling in moderation are all able to supply the demand for exercise.

Treatment laid down on the above lines, followed out in every instance, with good habits of hygiene and a careful observance of Nature's demands, will regulate the various functions of the body, and the menstrual function will prove no exception to the rule.

The following cases will substantiate the above:

CASE I. Miss C. S. K.—Seventeen years old. Decidedly anemic and much troubled with constipation. First menstruated at fourteen, since which time she has never been regular, flowing profusely sometimes twice a month, and other times going three or four months without menstruating at all. Has frequent fainting spells and a decided anemic heart murmur. At time of coming under observation had not menstruated for two months and ten days.

Treatment consisted of a regulated diet, tablets of aloin, strychine, belladonna and cascara sagrada, one each evening until bowels are regular, and teaspoonful doses of pepto-mangan (Gude) after meals. Gradually the fainting spells and heart symptoms disappeared, and on the fifteenth day after commencing treatment she began to menstruate, the flow being natural in quantity and continuing four days. Treatment was continued and twenty-nine days later she menstruated again, continuing this time five days. Soon after this the pepto-mangan was stopped. From now on, up to the present time, a period covering three months, her menses have appeared regularly every twenty-eight days.

Her whole appearance is changed and in every respect she appears well and strong. Period of administration of peptoman 55 days.

CASE II. Miss K. M.—Aged twenty. Menstruated first at age of fifteen and was fairly regular for three years, but since an attack of typhoid fever two years ago, has never known when she was going to be unwell. Patient was not thin, but face was pale and yellowish, hands and feet were cold "all the time," and her whole condition was one of "*blood poverty*." Complained of frequent attacks of diarrhœa following constipation.

Treatment consisted of plenty of out-door exercise, good food with abundance of milk, and pepto-mangan (Gude) in teaspoonful doses after meals.

Her restoration to health has been rapid and satisfactory. She has menstruated three times since beginning treatment, the longest interval being thirty-one days. Says she is all right and her appearance certainly sustains her words.

In this case the administration of pepto-mangan covered a period of thirty-six days.

CASE III. Miss D. L.—Schoolgirl. Aged fourteen. For two years she had been troubled with headaches, dizziness and short breath, fainting away at the slightest provocation. Had no appetite and as her mother expressed it, "for the last six months had been going down hill pretty fast." Had been treated by a physician for heart disease, but received no benefit. Menstruated first seven and a half months ago, "but had not seen anything since."

Examination showed heart to be normal, although it was a trifle fast, and a slight murmur could be determined when patient was in a recumbent position, evidently anemic in origin. Lungs proved to be all right.

Her general condition was anemic and she was put on pepto-mangan (Gude), a teaspoonful after meals, and sent into the country where she could be out doors most of the time and have plenty of eggs and milk. A letter from her mother says that she has changed so that she can hardly believe it is the same girl. Furthermore, her menses appeared twenty-one days after starting the pepto-mangan and returned again twenty-nine days after. The pepto-mangan was ordered stopped and since then I have not heard direct from the patient, although from her father I learn that she is "perfectly well" and coming home soon.

Period of administration of pepto-mangan, fifty-six days.

CASE IV. Miss L.—Aged eighteen. Had never menstruated. Her general appearance was one of profound anemia. A careful examination eliminated any abnormality of genital apparatus. Organs normal in relation, but undersized. Prescribed pepto-mangan in teaspoonful doses after meals and gave general directions as to diet, etc. Began to menstruate thirty-two days after beginning treatment, the flow continuing one week. Twenty-nine days later she menstruated again. At the present writing she is still under treatment and is due to menstruate in seventeen days. Her whole condition is very much improved.—*Vermont Medical Monthly, August 1897.*

